# Understanding Australian adolescent girls’ use of digital technologies for healthy lifestyle purposes: a mixed-methods study

**DOI:** 10.1186/s12889-022-13869-4

**Published:** 2022-08-01

**Authors:** Kate Parker, Laura Gould, Meenal Nand, Jonathan C. Rawstorn, Ana Maria Contardo Ayala, Ralph Maddison, Kim Toffoletti

**Affiliations:** 1 School of Exercise and Nutrition Sciences, Deakin University, Institute for Physical Activity and Nutrition (IPAN), Geelong, 3220 Australia; 2 School of Exercise and Nutrition Sciences, Deakin University, Geelong, 3220 Australia; 3 School of Humanities and Social Sciences, Deakin University, Geelong, 3220 Australia

**Keywords:** Technology, Social media, Teenager, Female, Physical activity, Body image

## Abstract

**Background:**

Little is known about girls’ use of a broad range of digital technologies to support a healthy lifestyle, particularly during the later adolescent years when they are expected to take greater responsibility for individually managing their health and wellbeing. The present study was designed to gain an in-depth understanding of adolescent girls’, 15 to 17 years of age, use of a broad range of digital technologies to support multiple healthy lifestyle purposes.

**Methods:**

This study used a mixed-methods sequential research design (i.e. quantitative and qualitative data in two consecutive phases). A quantitative online survey was conducted to determine the use of digital technologies for healthy lifestyle purposes. Qualitative semi-structured interviews were conducted with a subset of survey respondents to explore factors influencing their technology use and preferences.

**Results:**

Descriptive analysis of survey data (online survey, *n* = 336) demonstrated the popularity of social media and online video sharing platforms, with evidence that the use of digital technologies does not occur in isolation and girls draw on several resources simultaneously to achieve their desired healthy lifestyle outcomes. Thematic analysis of interview data (semi-structured interviews, *n* = 29) revealed seven interrelated themes around factors influencing girls’ use of, and satisfaction with, different digital technologies: accessibility, social connectivity, credibility, relatability, inspiration, safety, and customisability.

**Conclusions:**

The findings reiterate that one size doesn’t fit all girls, and often one size might not even fit one girl, and thus highlight the challenge of catering to such varied use cases. Overall, it appears that current digital technology offerings meet the needs and preferences of adolescent girls for healthy lifestyle purposes, however there may be benefit in allowing greater customisation of use, and consumer driven tailoring of content, according to personal preferences and changing circumstances.

**Supplementary Information:**

The online version contains supplementary material available at 10.1186/s12889-022-13869-4.

## Introduction

Adolescence is a life stage characterised by rapid changes in physical development, cognitive growth and social transitions [1] and is also an essential phase for establishing healthy habits that set the foundations for future health and wellbeing [2]. Indeed, increasing rates of chronic disease risk factors during adulthood can be attributed to a high prevalence of unhealthy lifestyle behaviours during adolescence [3]. For example, 81% of adolescents worldwide fail to achieve the recommended levels of physical activity for optimal health and wellbeing [4], majority (68%) of waking hours are spent sitting [5], as much as 72.7% sleep less than the recommended 8 h per night on average [6] and as few as 14% meet the recommended guidelines for vegetable intake [7]. The health and wellbeing of adolescent girls is of particular concern with higher rates of mental ill health [8], body image issues and disordered eating [9], and lower rates of physical activity [10], compared to their male counterparts, and these are more prevalent with increasing age throughout the adolescent years (i.e. 15–17 years compared to 12–14 years) [9]. Longitudinal research has also shown that the majority of adolescent girls either decrease or remain consistently inactive from adolescence through to young adulthood, with a very small proportion increasing their physical activity levels [11]. These patterns can be understood relative to changing societal understandings of adolescence as a period in which young people are encouraged to take personal responsibility for their future trajectories, including health outcomes, amidst competing priorities [12].

Societal influences on adolescents have never been more prominent than in today’s digital age, and the WHO-UNICEF-Lancet Commission on child and adolescent health [13] has called for a better understanding of how digital technologies, such as social media platforms, can positively engage adolescents about health issues. Researchers have increasingly explored the role of information and communication technologies (ICT) to deliver health promotion initiatives [14, 15] due to the potential for large population reach [16] and ubiquitous use and reliance on digital technologies (e.g., smartphone applications [apps], websites, social media and text messaging) among young people [13, 16–18], as well as the benefits observed when used for healthy lifestyle purposes [19–22], and cost effectiveness of intervention delivery [23]. However, evidence has consistently demonstrated poor long-term engagement – and a lack of lasting behaviour change [24] – which has largely been attributed to a failure to design interventions that satisfy adolescent girls’ preferences and needs [25].

To overcome this, studies have begun exploring how and why girls use digital technologies for various healthy lifestyle purposes (e.g., physical activity and sedentary behaviour, diet/eating, and to access healthy lifestyle information). For example, Goodyear, Boardley et al. [26] found that women’s (*n* = 69) use of social media platforms (e.g., Instagram) to inform their health-related attitudes, knowledge and behaviours centred mostly around influencer and celebrity stories and selfies with an emphasis on body appearance. Larson et al. [27] noted that adolescents (31 girls, 18 boys) recognise fitness apps (e.g., MapMyRun, Nike Run, PopSugarActive®) could support physical activity but lack social and community aspects of face-to-face sport and can perpetuate body image stereotypes that could be counterproductive for girls’ motivation [27]. Typically, studies have been designed to examine girls’ engagements with one particular technology for healthy lifestyle purposes, for instance, mobile apps [28], wearables [29] or social media [30–33]. Yet, little is known about girls’ use of a broad range of digital technologies to support a healthy lifestyle. There also remains a lack of exploration regarding how adolescents use digital technologies to address multiple unhealthy lifestyle behaviours [34]. Therefore, this study aimed to gain an in-depth understanding of older adolescent girls’ use of a broad range of digital technologies to support multiple healthy lifestyle purposes.

## Materials and methods

### Study design

This study used a mixed-methods sequential research design (i.e. quantitative and qualitative data in two consecutive phases). A quantitative online survey was conducted to determine the use of digital technologies for healthy lifestyle purposes (e.g., physical activity, healthy eating, social connections). Qualitative semi-structured interviews were conducted with a subset of survey respondents to explore factors influencing their technology use and preferences (i.e., likes and dislikes). Ethical approval was obtained from Deakin University’s Human Ethics Advisory Group – Health (HEAG-H 32_2021) prior to commencement of the study.

### Participants and recruitment

Eligible participants were females aged 15–17 located in Australia. Australian girls were the focus of the study because the research team was based in Australia and little is known about this cohort. Participants were largely recruited through 4 weeks of paid social media advertising across Facebook and Instagram targeting the eligible demographic. We also contacted a range of companies, Facebook groups and pages, influencers and websites that were likely to have a high following of eligible individuals with the request to promote the online survey. Out of 81 messages sent, six agreed to promote our survey link. In total, there were 1629 clicks on the link to the survey. Following this, informed consent from both the parent/guardian and adolescent was gained, (adolescents and parents were asked to provide electronic consent by ticking a box on the online consent form) and the survey was completed. Survey completers who consented to be contacted for an interview (*n* = 138) were invited via email and/or a phone call to participate.

### Data collection

#### Online survey

The online survey was conducted via the Qualtrics survey platform (https://www.qualtrics.com) during May 2021. Potential participants were asked to self-report their gender (female, male, other), age (14 years or younger, 15, 16, 17, 18 years or older), and residential postcode to determine eligibility. Eligible and consenting participants were asked to indicate their use (current, previous, never) of social media (Instagram, Facebook, TikTok) and online video sharing platforms (YouTube), purpose-built mobile apps (e.g., Strava, Keep It Cleaner, JSHealth Nutrition) and websites (e.g., Blogilates), wearable technologies, and live delivery platforms (e.g., Zoom, Skype) for healthy lifestyle purposes. Participants were informed that ‘healthy lifestyle purposes’ referred to using the technologies to assist or guide them to be more physically active, sit less, increase social connections, improve sleep or eat healthy. Participants who reported current use of a particular technology were asked to self-report their reasons for use (increasing physical activity or exercise, increasing fitness level, reducing sitting time, increasing social connections, improving sleep, eating healthily, other), frequency (days per week) and total length of use (< 1 month, 2–6 months, 6–12 months, > 12 months), and where relevant, the names of specific social media and video sharing accounts, apps, websites, wearables and platforms they use.

#### Semi-structured interviews

Semi-structured, individual interviews were conducted online via the video conferencing platform Zoom throughout June–July 2021 by two authors (LG and MN). Interviews ranged between 18 and 36 minutes. Question development was guided by the overarching research question ‘What factors influence adolescent girls’ use and preferences of digital platforms for health and wellbeing?’ and the Unified Theory of Acceptance and Use of Technology (UTAUT) framework [35]. UTAUT theorises that performance expectancy (e.g., usefulness, extrinsic motivation, outcome expectations), effort expectancy (e.g., perceived ease of use or complexity), social influence (e.g., social norm), and facilitating conditions (e.g., perceived behavioural control and compatibility) are the direct determinants of acceptance and use of technology [35]. Participants were asked about their reasons for using, and satisfaction with, different digital technologies to support healthy lifestyle changes. An interview guide (see Additional file 1) was used to initiate discussion, ask follow-up questions, and maintain consistency across interviews. Interview conversation focused on 5 topics: (i) healthy lifestyle; (ii) functionality and ease of use; (iii) quality of content/information; (iv) social norms and influences; (v) preferences. Participant survey results provided the basis from which to tailor interview discussions around most relevant digital technologies and healthy lifestyle behaviours. Interviews were recorded in Zoom, downloaded, and transcribed verbatim by a university approved transcription provider.

### Data analysis

STATA v16 [36] was used to calculate descriptive statistics for sample characteristics and use of digital technologies for healthy lifestyle purposes. Content analysis was used to synthesise the social media and video sharing accounts, apps, websites, wearables, and platforms which participants currently engaged with for healthy lifestyle purposes. Content analysis enables systematic and objective inferences from text data to quantify and describe the manifest and latent content in context [37]. LG created a codebook in Excel to categorise the types of social media and video sharing accounts, apps, websites, wearables, and platforms participants were currently using after reviewing all participant responses. Two additional authors (KP and MN) reviewed this codebook and discussions were held until consensus was reached. Each of these three authors (KP, MN and LG) then independently coded all participant responses and further discussions were held to establish agreement regarding the final coding of responses.

Qualitative interview data were analysed thematically (inductive, semantic) following Braun and Clarke’s [38] six phase approach: (i) familiarisation; (ii) coding; (iii) searching for themes; (iv) reviewing themes; (v) defining and naming themes; (vi) reporting. The theoretical flexibility of thematic analysis [38] made it a suitable content analysis method for our inductive approach. Data familiarisation occurred through transcription of interviews and reading and re-reading of transcripts. To ensure trustworthiness and accuracy, multiple members of the research team were involved in making coding, analysis and interpretive decisions. The process of identifying codes and constructing themes was jointly undertaken by authors KT, KP, MN and LG to ensure rigor. This process involved assigning codes to interview excerpts, grouping codes under common themes, and assigning names to each theme. NVivo software was used to code and organise the data.

## Results

In total, 336 participants completed the online survey (completion rate = 20.6%). Just under half (44%) were 17, 32% were 16 and 24% were 15 years of age. The majority lived in major cities (75%), while 21% lived in inner regional areas and only 4% lived in outer regional or remote areas. A larger proportion lived in areas of high socio-economic status (30%) compared to low socio-economic status (11%).

### Quantitative findings

**Fig. 1:**
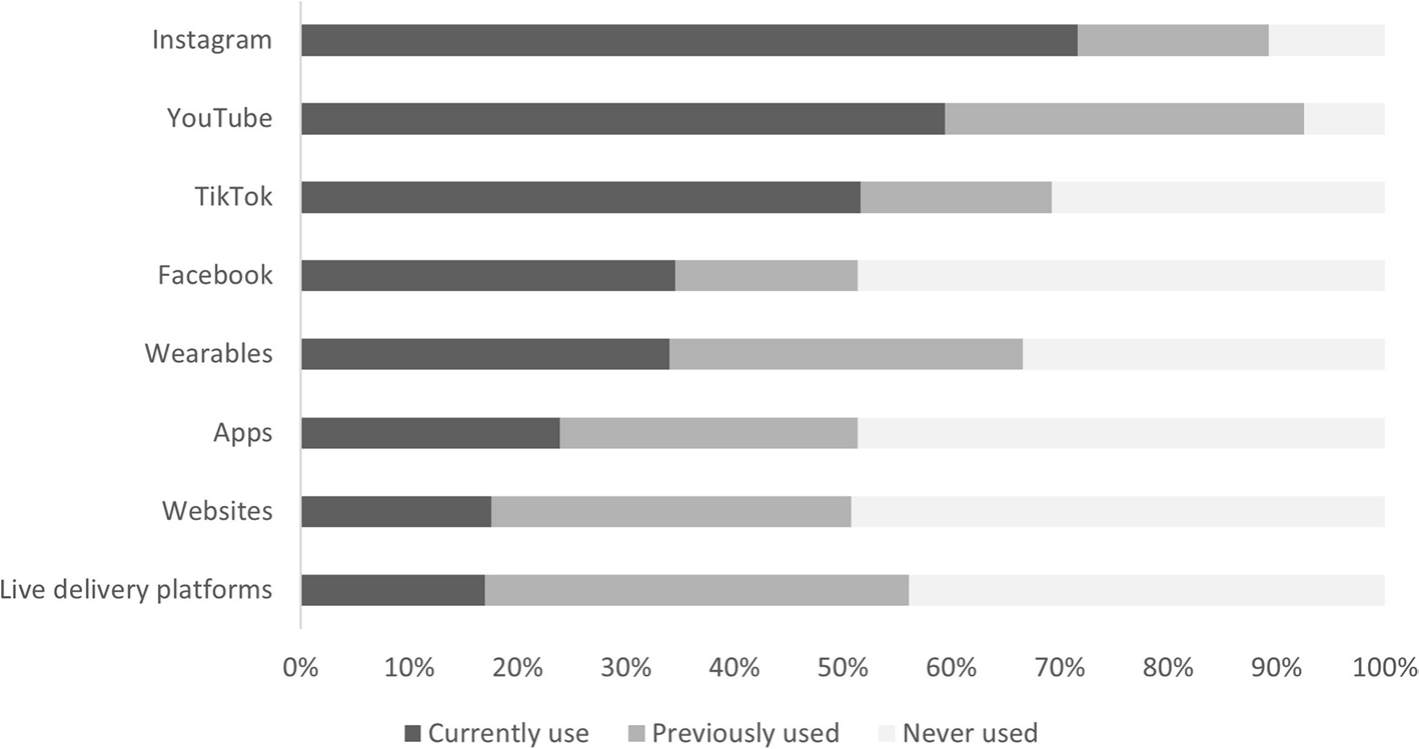
Proportion of participants currently, previously, and never using each digital technology for healthy lifestyle purposes

The three most common technologies currently being used for healthy lifestyle purposes were Instagram (72%), YouTube (59%) and TikTok (52%). Live delivery platforms were cited as previously used by 39% of participants, however, just 17% were currently using them (Fig. 1).

Table 1 shows that majority of participants who currently used YouTube (75.9%), wearables (87.7%), apps (73.8%) and websites (54.2%) did so to increase physical activity or exercise, and the majority who used wearables did so to increase their fitness level (81.6%) and/or reduce sitting time (64.9%). Instagram (78.8%) and Facebook (79.1%) were most commonly used for increasing social connections. Just under half of participants who used wearables (48.3%) and apps (42.5%) reported doing so to improve their sleep. More than half of girls who used apps (60.0%), websites (54.2%), Instagram (51.3%) and YouTube (50.3%) did so for healthy eating purposes. Supplementary Tables 1 and 2 (see Additional files 2 and 3) provide an overview of the extent of use of each digital technology.

**Table 1:** Proportions of participants currently using each digital technology for different healthy lifestyle purposes, n (%)

	Instagram *n* = 240	YouTube *n* = 199	TikTok *n* = 173	Facebook *n* = 115	Wearables *n* = 114	Apps *n* = 80	Websites *n* = 59	Live delivery platforms *n* = 57
Increase physical activity or exercise	79 (32.9%)	151 (75.9%)	66 (38.2%)	12 (10.4%)	100 (87.7%)	59 (73.8%)	32 (54.2%)	14 (24.6%)
Increase fitness level	67 (27.9%)	95 (47.7%)	52 (30.1%)	8 (7.0%)	93 (81.6%)	46 (57.5%)	26 (44.1%)	13 (22.8%)
Reduce sitting time	17 (7.1%)	21 (10.6%)	19 (11.0%)	4 (3.5%)	74 (64.9%)	24 (30.0%)	9 (15.3%)	7 (12.3%)
Increase social connections	189 (78.8%)	40 (20.1%)	88 (50.9%)	91 (79.1%)	15 (13.2%)	17 (21.3%)	12 (20.3%)	25 (43.9%)
Improve sleep	29 (12.1%)	52 (26.1%)	34 (19.7%)	5 (4.4%)	55 (48.3%)	34 (42.5%)	21 (35.6%)	4 (7.0%)
Eat healthily	123 (51.3%)	100 (50.3%)	67 (38.7%)	23 (20.0%)	31 (27.2%)	48 (60.0%)	32 (54.2%)	9 (15.8%)
Other	31 (12.9%)	25 (12.6%)	41 (23.7%)	19 (16.5%)	5 (4.4%)	10 (12.5%)	14 (23.7%)	14 (24.6%)

Purposes not mutually exclusive

Table 2 represents the top five most common types of social media and online video sharing platforms, purpose-built apps and websites, wearable technologies, and live delivery platforms that participants currently engaged with for healthy lifestyle purposes. On the three social media platforms (Instagram, TikTok and Facebook), participants mostly viewed their friends, family and general feeds for healthy lifestyle purposes. Participants used YouTube and purpose-built apps most commonly for fitness, workouts, and exercise, whereas websites were most used for nutrition, food, and recipes with half as many participants reporting use of exercise-based websites. Apple Watch and Fitbit were the most used wearables, and Zoom was the most used live delivery platform.

**Table 2:** Top five types of technologies participants engaged with for healthy lifestyle purposes

	Instagram	YouTube	TikTok	Facebook	Wearables	Apps	Websites	Live delivery platforms
1	Friends, family & general feed (31%)	Fitness influencers (40%)	Friends, family & general feed (32%)	Friends, family & general feed (18%)	Apple Watch (46%)	Exercise (36%)	Nutrition, food, recipes (23%)	Zoom (55%)
2	Nutrition, food, recipes (15%)	Workouts only (12%)	Nutrition, food, recipes (17%)	Local/community groups (17%)	Fitbit (41%)	Nutrition, food, recipes (23%)	Health information (17%)	Teams (13%)
3	Fitness influencers (11%)	Healthy lifestyle influencers (10%)	General influencers, celebrities (14%)	Nutrition, food, recipes (16%)	Garmin (7%)	Combined exercise and nutrition (12%)	Miscellaneous (15%)	Skype (13%)
4	Healthy lifestyle influencers (8%)	General influencers, celebrities (10%)	Fitness influencers (12%)	Miscellaneous (16%)	Samsung Watch (3%)	Mindfulness, meditation, mental health (8%)	Combined exercise and nutrition (11%)	Google Meet (5%)
5	General influencers, celebrities (8%)	Nutrition, food, recipes (9%)	Miscellaneous (11%)	Sport, personal trainer, club specific page (11%)	Fossil Watch (2%)	Miscellaneous (7%)	Exercise (11%)	Facetime (5%)

### Qualitative findings

A total of 29 survey participants were interviewed (completion rate = 21%). Analysis of interview data identified seven interrelated themes describing factors that influence girls’ use of, and satisfaction with, different digital technologies for healthy lifestyle purposes: accessibility, social connectivity, credibility, relatability, inspiration, safety, and customisability (see Additional file 4 for codes).

#### Accessibility

Participants favoured digital technology that was accessible in terms of (i) cost: *‘I’d rather just use the free content instead of pay for it*’; and (ii) time: short videos and infographics were considered *‘good if I just want to look at something quick or if I need advice’*, as were embedded links to longer content, which girls could pursue *‘depending on how much time I have’*. They also demonstrated a preference for digital technologies allowing easy sharing of content and access to different types of accounts (e.g., account holders who promote healthy food or workout tips): *‘I definitely like the freedom to be able to click what account suits your needs’*.

Girls also characterised accessibility in terms of relevance of content. They discussed wanting access to digital content that represented people of different body types, offered content appropriate for their age and skill level and designed for different fitness levels: *‘if you provide a workout, provide a beginner version, an intermediate and an advanced’*. Some participants who relied on social media for healthy lifestyle content sought more support in determining digital resources to access: *‘a bit of guidance would be good, so maybe a quiz or something, when you download the app. Like, what do you want to achieve? What are your goals?’*

#### Social connectivity

The ability to connect socially (comment, follow, like, share, watch, link) online was important for participants, although the preferred amount of connection varied. Some avoided livestream technologies (e.g., Zoom or Instagram live) to maintain privacy and anonymity, others did so by minimising interaction on public accounts/forums (e.g., did not comment on or ‘like’ content they were consuming). Other girls described using platforms and apps with comments and closed group functions to connect with like-minded people and communities: ‘*I’ve made friends where we...started by sharing recipes’*. Girls liked technologies that allowed them to easily message workout and recipe recommendations to friends (e.g., TikTok and Snapchat), and which provided access to public accounts and forums to obtain advice and ask questions: *‘sometimes I’ll leave a comment, maybe complimenting the recipe or maybe asking – because I’m gluten-free – “Can I use maybe gluten-free flour for this?”’*. Responses indicated that girls felt supported in their health efforts by interactive online engagements: *‘we send each other posts – I mean, healthy lifestyles is also about communication, having connections and stuff, so I think I connect with most of my friends on Instagram so I can have – there’s a support system’*. Girls articulated the importance of connecting with peers in ways that maintained privacy and safety - they did not always trust social media platforms to provide this and articulated a desire for different options:

*I think it would be really helpful to have a communication portal, like a chat or something, where you can just put anonymous questions and get any answers you want. I think it would be really important to filter the answers and have a blocker on certain key words, but I think it would be really good to just be able to communicate with other people your own age, without having judgement there with it.*

#### Credibility

On being asked to reflect on the quality of fitness information encountered on digital platforms and apps, girls discussed the credibility of sources and the importance of health content that was *‘factual’* and based on *‘scientific evidence’*: *‘if any influencer is allowed to just post whatever they want, put up whatever they want, it can become not so good’*. Social media’s public and user-generated content model allows anyone to post health information. In this context, girls were not always certain about how to determine credible online health sources:

*I feel the problem with workouts on Instagram is, when people post, but then you don’t know whether they’re giving genuine advice or they’re just like an influencer who’s trying to market some product or something towards them because they have a sponsorship with someone. It gets a bit confusing about who’s actually also giving the real facts.*

Participants wanted greater certainty that healthy lifestyle information and tips accessed online and via apps was accurate and safe:

*Have moderators on there. Certified nutritionists or PTs and stuff like that, and not just amateurs kind of posting whatever they want. Because that means you can actually fact check the information and it won’t be harmful to certain people that might have triggers.*

#### Relatability

Girls described being drawn to digital content they could relate to. For some participants, relatable resources were those created and/or circulated by peers: *‘If they’re close to my age then I am more likely to watch them’*, and those which felt *‘genuine’* and achievable: *‘she also posts workouts and recipes which is good, because she’s also a teenager. She doesn’t have that much experience cooking, so if she can make the recipes, I can too’*. Other girls related to health and fitness content that shared personal stories: *‘the people that I follow, they’re not really giving facts. It’s more they’re showing me their journey, and I can tell it’s authentic’*. Modes of personal address generated feelings of connection between girls and the people they followed for health advice, inspiration, and entertainment. But participants expressed the preference for personal experiences to have some basis in *‘fact’* and disliked content that they believed made *‘false promises’*: *‘there’s a lot of, “If you do this diet for a month, your body will become from this to the perfect image.” And I’m like, “I don’t think that’s really real. It doesn’t seem realistic”’.*

#### Inspiration

Participants articulated a strong preference for digital encounters that supported and encouraged healthy behaviours *‘like, “keep going”’* over prescriptive instructions *‘like, “Do 10 burpees”’.* Some discussed using tracking apps/devices to keep them accountable through goal setting and prompts, with girls identifying features such as challenges, trophies and monitoring (e.g., heart rate) as helpful for achieving this: *‘it’s good to see the progress you’ve made, so it gives you motivation to continue doing it’*. Mood trackers were viewed positively: *‘it helped with mental health’*, while calorie counters were viewed negatively: *‘all the stuff about the counting calories, is probably not necessarily good.’* Key sources of motivation included guided audio and video, and social media accounts: *‘I normally look at them and inspiration and stuff for ways to inspire me because I do struggle to get active sometimes’*. Girls reported feeling *‘self-conscious’* and *‘vulnerable’* when exercising on live delivery platforms and expressed preferences for digital technologies and content that inspired them to exercise at times when they *‘can’t really be bothered’: ‘looking at YouTube for inspiration for your workouts, and then you do them by yourself afterwards’*. *‘Fun’ *and *‘new ideas’* were important to participants to capture their interest, maintain engagement and find motivation:

*If it’s just do ten push-ups and then do some arm workout and stuff I’m not going to be appealed to do it but if it’s like, do this or do a dance that’s fun and engaging and involves other aspects of my life, like the rollerblading I find really helps me keep in shape because I think it’s fun*.

#### Safety

Safety was an important element in teenage girls’ decision making around what digital technologies to use and how they used them for healthy lifestyle behaviours. Girls conceptualised safety in various ways. Fear of judgement made girls feel unsafe, and was a key factor shaping girls’ platform use: *‘putting out your own opinion out there is quite challenging because a lot of people might want to bring it down or counteract you’*, along with pressure to meet unrealistic fitness/body expectations:

*when you go on Instagram or these platforms where you see people, it’s constantly showing the best image of this person, and showing they’re the best that they can be kind of thing, and instead of making me feel better most of the time, it just makes me feel “oh I’ll never be able to achieve that”*.

Girls expressed dissatisfaction with the limited range of body types promoted in health and fitness content and wanted alternatives to support psychological safety and positive body image: [there’s a] *‘really big emphasis to achieve a certain body type because obviously everybody’s bodies are different and it is sometimes really difficult for one person to achieve one type. Maybe a variety of options’*.

Toxic digital environments were an aspect of social media that undermined users’ feelings of safety:

*toxic as in a lot of people just edit their photos. It’s not very real ... they could be promoting harmful products or just products that don’t work and then getting money off that, and they’re just kind of manipulating their followers which is very – I think it’s very toxic of the creators*.

‘Toxic’ also referred to cyber bullying/nastiness. Therefore, features on social media platforms that supported girls to avoid (block, report, unfollow) inappropriate accounts, suggested topics and negative or hostile comments were considered important: *‘you can click on things and say that you’re not interested in a certain thing or unfollow people and make sure that they don’t appear again’*. As alluded to earlier, participants appreciated platform features that enabled privacy and anonymity: *‘you don’t have to put your actual name, so you can just hide behind your TikTok user-name if you’re scared that people don’t get your information’.*

#### Customisability

Results indicate girls’ desire for more customisable online health content. They measured the effectiveness of digital resources for promoting healthy lifestyle behaviours in terms of ability to minimise negative content and maximise supportive, credible, safe and accessible content. Participants sought a personalised experience that suited their lifestyle (e.g., rollerblading or dancing): *‘a variety of workouts, I think I would like to see. Like a kind of platform where you can search up what you’re feeling and they’ll give you a really fun workout’.* They also wanted freedom to choose when to access healthy lifestyle content: *‘I want to choose my own time to do things. So, “This class will be scheduled for 7:00pm,” and I’m like, “What if I want to do it at 8:00pm?”’.* Participants using social media for healthy lifestyle purposes expressed an awareness of the workings of algorithms to determine the content they see and sought greater autonomy in decision-making around content preferences:

*If there was a way to customise it. Because I know I want to see all these models and stuff, but at the same time, I know it’s not good for me. So if there was a way to fix that*.

Girls reported challenges in managing their exposure to digital content in terms of (i) time: *‘I’ve kind of forced myself to avoid them, like sometimes even putting timers on these apps so I can’t use them more than a specific time within a day’* and (ii) volume of online content, which girls described as *‘distracting’* and *‘overwhelming’*. Features that allowed girls to customise digital content for healthy lifestyle purposes included the ‘save’, ‘filter’ and ‘favourite’ functions: *‘I really like the save button, like you can save posts and you can organise them’*. These features helped girls to easily locate preferred content, minimise unwanted content and/or download for later use: *‘you can put them into little collections, so it’s really easy to find’.*

## Discussion

This study illustrates the importance of a mixed-methods approach to gain an in-depth understanding of Australian older adolescent girls’ (aged 15–17 years) use of a broad range of digital technologies to support multiple healthy lifestyle purposes. The findings demonstrate that the use of digital technologies does not occur in isolation and that girls draw on several resources simultaneously to achieve their desired healthy lifestyle outcomes.

Girls’ technology use and preferences were influenced by a desire for greater control over levels of social connection and online visibility. Research has indicated that adolescent girls value feeling connected to, and supported by, individuals and social groups in their healthy lifestyle efforts [39]. It is suggested that these feelings of support and connection are strongest when girls and women develop and maintain groups where participants are known to each other or interact around topics of shared interest [26, 32]. This may explain the high use of social media (Instagram, Facebook and TikTok) and the low uptake of apps for physical activity among survey participants, as apps remove key social and interactive aspects that girls value [28]. Use of social media for social connections is also unsurprising as this study was conducted when parts of Australia were coming out of lockdown restrictions due to the coronavirus (COVID-19) pandemic and social communications frequently occurred via digital technologies [40]. However, our findings add new insights to understanding the digital engagements that that shape girls’ preferences. Qualitative interviews suggest some girls’ achieved feelings of social connection simply by looking at (scrolling, searching, clicking links) publicly available health content/accounts that they felt they could relate to. Feeling connected digitally does not require real time presence, as indicated by the limited use of live delivery platforms (e.g. Zoom) by girls for healthy lifestyle purposes (however a greater proportion had previously done so, therefore it is possible that girls were harnessing these technologies during lockdowns but found little ongoing use for them when in-person opportunities became available). Participants reported being drawn to technologies where they could find content produced by young women that they perceive a likeness to. These forms of social connection enabled girls to bypass interactions that could subject them to negative judgement, scrutiny and critique, and fostered feelings of safety by allowing the choice of anonymity when consuming health related content. Like Toffoletti’s (et al.) [41] study with adult women, maintaining privacy and safety were important considerations, yet girls did not select digital health technologies on this basis. Rather, they altered their social media practices by minimising interactivity and therefore ‘visibility’ (i.e., not posting or commenting or liking), but felt they were still able to participate socially by scrolling through content. This suggests a need for enhanced capabilities for anonymity when engaging with digital technologies for healthy lifestyle purposes for this cohort.

With respect to the role of social influence as a factor influencing girls’ digital use, this study adds new insights into the value of digital technologies that deliver relatable, credible, and non-judgemental content to support healthy lifestyles. Whereas past research has emphasised the role of celebrity influencers as sources of motivation and knowledge for girls’ healthy lifestyles [19, 31, 42], interviews with older adolescent girls highlighted a high degree of critical awareness of the tactics used by influencers to claim expertise and establish trust by cultivating feelings of connection and intimacy with users. Accordingly, participants questioned the evidence-base of information (apps, accounts, programs) produced by influencers, and attributed higher value to technologies that allowed them to draw on each others' food and fitness experiences as sources of information and inspiration they could relate to, and they felt were achievable. Girls’ preferences for digital health technologies and content that promoted realistic experiences and expectations meant that they often considered their peers as sources of credible knowledge. This indicates that credibility is assessed differently by girls depending on the source of information (e.g. relatability of peer versus experience of fitness influencer) and led to girls expressing uncertainty at times around how to determine a credible source.

Our findings show adaptive content curation features that allow girls to limit unwanted content and promote preferred content were a key driver for digital technology uptake, and adolescent girls are highly skilled at navigating complex and contradictory messaging on social media regarding healthy lifestyle behaviours [32, 33]. Supporting existing studies [32], qualitative data highlight adolescent girls’ competence and creativity in managing challenging aspects of digital health technologies, such as unrealistic (e.g., ‘perfect’ bodies) and directive (rather than encouraging) messaging which contributed to feeling intimidated and/or discouraged. Girls demonstrated a critical understanding of ‘perfect’ bodies as ‘fake’, ‘toxic’ and unrealistic, therefore not relatable and demotivating. While a study of younger adolescent girls (13–15) found they used health related social media to post pictures of themselves to friends [32], discussions with the older adolescent girls (15–17) in our cohort suggested they used social media mainly to share content produced by others (e.g., influencer workouts) and peer content related to experiences. They expressed reservations about showing off their bodies or opinions online, indicating instead that they used digital technologies to seek out and circulate information, share achievements, and provide and obtain support to maintain healthy lifestyle practices.

There was evidence that girls understood which content was not ‘good for them’, and used these types of curation features to increase exposure to ‘appropriate’ content, which they defined as fun, achievable, safe, credible, and suitable to their levels of skill and competence. In light of these findings, we suggest digital products give users more control than what is offered by current features (i.e., up/downvoting, liking and subscribing) to determine what content they are shown (e.g., option to select ‘I don’t want to see this’, ‘more like this’, ‘not relevant’) – to support girls in navigating platform algorithms designed for particular demographics and lifestyles goals.

A new finding from this research is that girls intuitively developed customisation strategies to proactively manage large amounts of health-related digital content. Girls discussed that drawing content from video platforms like YouTube, following multiple social media accounts, using apps and wearables to support their healthy lifestyle could lead to information overload and spending more time on digital devices than they wanted. Participants reported preferring no-cost technologies that are free to sign up and allowed them to easily access, download, store and share content, such as YouTube videos or recipes they could file for later use. This supports survey findings showing that social media and online video sharing platforms were used most frequently by girls for healthy lifestyle purposes compared to purpose-built apps, websites, wearables, and live delivery platforms with less functionality for saving and sharing. Low uptake of wearables, apps and live sessions may be attributed, in part, to cost and time factors, as girls liked sharing free resources with each other and welcomed the flexibility of selecting workouts that fitted in with their schedules. This might explain why only a small proportion of girls in this sample were currently using purpose-built apps and wearables, however those that did appeared to use them for a multitude of reasons (including physical activity and exercise, fitness, healthy eating, reducing sitting time and sleep). Previous research has demonstrated limited long-term engagement with purpose-built apps and wearable devices among adolescents [43, 44], with focus groups highlighting concerns regarding the effort of use, risks regarding damage to or loss of the device and compatibility with daily life [45]. Downloading preferred content on their personal devices allowed girls to retain and easily access content from a variety of platforms, that suited their levels of ability, needs and preferences. In light of evidence that girls are reluctant to undertake healthy lifestyle activities they consider above their level of ability (i.e., too difficult) [31], we recommend exploring tools/features that support older adolescent girls to customise vast amounts of available information, and resources to support them in determining and evaluating the content that is best suited to support their goals and circumstances (recognising that these will likely change over time). Participants also called for more access to content showing diverse body types, realistic body shapes and options for different levels of ability. The latter features are typical of apps, though only a small proportion of girls in this sample were currently using purpose-built apps and wearables due to cost, limited shareability and opportunities for social engagement [28].

This study identifies several implications for practitioners and policymakers. The concerns raised by the young women in our study are not considered in the Australian government’s rating systems for ‘healthy living apps’ (https://www.vichealth.vic.gov.au/media-and-resources/vichealth-apps/healthy-living-apps), which measure app effectiveness only in terms of functionality and behaviour change. Government organisations should include social media sources that are most popular with adolescent girls and consider developing ratings to address their concerns around credibility, content diversity and safety. Future research could investigate the efficacy of existing resources for supporting girls’ decision-making around digital technologies for healthy lifestyle purposes. Given girls’ preferences, public health and other organisations with an adolescent girl audience should look towards increasing diversity in shareable content. Girls may also benefit from a credibility checklist that includes basic questions as prompts to think about how much credibility they want/need, to self-determine credibility of sources/creators as required.

There were limitations to the study. Survey sample size was limited, however interview data supported and added credibility to the findings. Participants were provided with a list of commonly used digital technologies among the target group, which may have restricted participant responses. For example, there may be other social media or online video sharing platforms that this target group engage with that were not listed and thus, this information could not be captured. It should also be noted that this study was conducted during the COVID-19 pandemic; use of digital technologies for healthy lifestyle purposes may have changed during this period among participants in some Australian jurisdictions in response to restrictions on public gatherings and recreational activities.

## Conclusion

Overall, the study findings demonstrate that Australian older adolescent girls are active engagers with social media and online video sharing platforms for healthy lifestyle purposes. To date, there remains a lack of research investigating the use of such platforms to promote positive health behaviour change in this target group. This study provides new evidence regarding older adolescent girls’ engagement with digital technologies for healthy lifestyle purposes. A key finding is that older adolescent girls employ a variety of different technologies for different health and lifestyle purposes and tend to download content to develop a customised suite of resources they can access from their personal devices that suit their needs and fit in with their schedules. While the collation of content was self-directed, girls continued to rely on social media, purpose-built apps and wearables for inspiration to motivate them to practice healthy lifestyle behaviours. The range of reasons for use and ways in which girls engaged with the platforms highlight the challenges of catering to varied use cases (i.e., no one size fits all). Results suggest that digital technologies, particularly social media apps, could consider integration of features that allow for consumer driven tailoring of content, ability for anonymity or invisibility in use, and assurance of credible yet relatable information. Subsequent research could investigate if the combination of several digital resources is objectively effective at improving healthy lifestyles of adolescent girls.

## Supplementary Information

**Additional file 1.** Interview guide.

**Additional file 2: Supplementary Table 1.** Length of time that participants used each digital technology for healthy lifestyle purposes.

**Additional file 3: Supplementary Table 2.** Number of days per week that participants used each digital technology for healthy lifestyle purposes.

**Additional file 4.** Themes and codes used in thematic analysis.

## Data Availability

To request data from this study, please contact Dr. Kate Parker: kate.parker@deakin.edu.au
