# Supplementary material for: Understanding Australian adolescent girls’ use of digital technologies for healthy lifestyle purposes: a mixed-methods study
Source: BMC Public Health. 2022 Aug 1;22:1464. doi: 10.1186/s12889-022-13869-4 (PMC9341407; doi:10.1186/s12889-022-13869-4)
Supplement: Supplementary file 1 — Additional file 1. Interview guide. [file 12889_2022_13869_MOESM1_ESM.docx]

Interview guide

| Topic | Key question | Importance |
| --- | --- | --- |
| Establishing parameters | How would you define ‘healthy lifestyle behaviour’? / What does ‘healthy lifestyle behaviour’ mean to you?  I notice that in the survey you reported using X. Could we talk a bit more about your use of this platform? |  |
| Healthy lifestyle | How does this platform/account help you to stay healthy, and do you think this platform has been effective to improve your lifestyle?  Do you feel like it’s been effective? How so?  In what ways does using this platform make it easier to have a healthy lifestyle, compared with if you were not using it?  What would encourage you to use this platform more often for health purposes? | How important do you think it is to use a digital platform to have a healthy lifestyle? |
| Functionality and ease of use | I’d be interested in hearing more about your experiences of navigating the platform and engaging with the content. Can you tell me about the features/functions that you like and use a lot?  Are there any features/functions you don’t like, or make it hard to use?  Are there any features/functions you would add if you had control?  Would you add a new feature/function? any example?  Are there any aspects of using this platform that worry you/ that you dislike? |  |
| Quality of content / information | How would you rate the quality of the information? (prompt for credibility, clarity).  Can you tell me what you like about the content?  Is there anything you don’t like about the content delivered on the site/app?  How easy is it to find the kind of content you are looking for? (Follow up: why do you think that is?) | How important is it to you that the information is accurate and credible / scientifically proven?  How important is it that it looks and feels engaging? |
| Social norms and influences | Can you tell me a bit about who else is using this platform/app (or following a particular influencer)? (prompt friends, other girls your age etc).  If others were not using or following it, would you continue to do so? Follow up: why/why not? | How important is it to you that other people you know are using it? |
| Preferences | What makes this platform/app/device better than other ones you have tried?  What makes X account more interesting/ engaging than others?  You stated that you have never used X, Is there a reason why you have never used this?  In the past you used x and said X was why you don’t anymore. Can you tell me a bit more about that? |  |
