# Supplementary material for: Understanding Australian adolescent girls’ use of digital technologies for healthy lifestyle purposes: a mixed-methods study
Source: BMC Public Health. 2022 Aug 1;22:1464. doi: 10.1186/s12889-022-13869-4 (PMC9341407; doi:10.1186/s12889-022-13869-4)
Supplement: Supplementary file 2 — Additional file 2: Supplementary Table 1. Length of time that participants used each digital technology for healthy lifestyle purposes. [file 12889_2022_13869_MOESM2_ESM.docx]

Supplementary table 1. Length of time that participants used each digital technology for healthy lifestyle purposes

|  | Instagram | YouTube | TikTok | Facebook | Wearables | Apps | Websites | Live delivery platforms |
| --- | --- | --- | --- | --- | --- | --- | --- | --- |
| Less than 1 month | 4.3% | 9.2% | 8.2% | 14.2% | 8.9% | 11.7% | 8.9% | 28.0% |
| 2 to 6 months | 16.6% | 21.4% | 24.1% | 22.1% | 12.5% | 22.1% | 21.4% | 14.0% |
| 6 months to 1 year | 17.5% | 18.9% | 27.7% | 22.1% | 19.6% | 16.9% | 21.4% | 26.0% |
| More than 1 year | 61.7% | 50.5% | 40.0% | 41.6% | 58.9% | 49.4% | 48.2% | 32.0% |
