# Supplementary material for: Understanding Australian adolescent girls’ use of digital technologies for healthy lifestyle purposes: a mixed-methods study
Source: BMC Public Health. 2022 Aug 1;22:1464. doi: 10.1186/s12889-022-13869-4 (PMC9341407; doi:10.1186/s12889-022-13869-4)
Supplement: Supplementary file 3 — Additional file 3: Supplementary Table 2. Number of days per week that participants used each digital technology for healthy lifestyle purposes. [file 12889_2022_13869_MOESM3_ESM.docx]

Supplementary table 2. Number of days per week that participants used each digital technology for healthy lifestyle purposes

|  | Instagram | YouTube | TikTok | Facebook | Wearables | Apps | Websites | Live delivery platforms |
| --- | --- | --- | --- | --- | --- | --- | --- | --- |
| 1 day per week | 6.0% | 25.9% | 10.2% | 26.6% | 1.8% | 9.0% | 29.6% | 51.0% |
| 2 days per week | 13.6% | 21.3% | 9.6% | 19.5% | 4.6% | 7.7% | 14.8% | 22.5% |
| 3 days per week | 13.2% | 19.8% | 16.3% | 13.3% | 2.7% | 6.4% | 22.2% | 12.2% |
| 4 days per week | 6.4% | 7.1% | 10.2% | 8.9% | 1.8% | 14.1% | 9.3% | 4.1% |
| 5 days per week | 11.5% | 11.2% | 10.8% | 7.1% | 6.4% | 15.4% | 3.7% | 2.0% |
| 6 days per week | 10.6% | 3.6% | 8.4% | 16.8% | 6.4% | 1.3% | 3.7% | 2.0% |
| 7 days per week | 38.7% | 11.2% | 34.3% | 8.0% | 76.4% | 46.2% | 16.7% | 6.1% |
