# Supplementary material for: Understanding Australian adolescent girls’ use of digital technologies for healthy lifestyle purposes: a mixed-methods study
Source: BMC Public Health. 2022 Aug 1;22:1464. doi: 10.1186/s12889-022-13869-4 (PMC9341407; doi:10.1186/s12889-022-13869-4)
Supplement: Supplementary file 4 — Additional file 4. Themes and codes used in thematic analysis. [file 12889_2022_13869_MOESM4_ESM.docx]

Themes and codes used in thematic analysis

| Themes | Codes |
| --- | --- |
| Accessibility | Content designed for different levels  Cost  Detailed content (longer videos)  Digestible content (shorter videos)  Easy to find relevant content  New content to meet changing needs and preferences  Share relevant content  Time  Wide variety of different perspectives |
| Social connectivity | Being visible on social media  Being invisible on social media  Communication  Community  Connection  Interaction  Peer information sharing  Relationship building  Sharing (others’ posts and user-generated content, information)  Talking to friends  Talking to users with shared interests  User engagement |
| Credibility | Misinformation or information  Number of followers  Reliability  Reviews  Trustworthy  Truthful, factual, evidence-based content  Verified ticked accounts  Verifying sources |
| Relatability | Authenticity  Experience  Factual content  False promises  Genuine  Unrealistic ideals |
| Inspiration | Can’t be bothered  Confidence  Encouragement and support  Having needs acknowledged  Motivation (and lack of)  Promoting critical engagement  Self-monitoring  Self-care |
| Safety | Avoiding pressure to meet performance, aesthetic and lifestyle expectations  Avoiding judgement  Body image  Inappropriate accounts  Online abuse/aggression/negative comments  Psychological safety  Toxic environment  Unrealistic expectations |
| Customisability | Autonomy in decision making  Awareness of algorithms  Effectiveness  Filter system  Freedom to choose  Managing files  Saving and downloading content  Self-selection of content  Wide variety of content to choose from |
